# The Recognition of and Care Seeking Behaviour for Childhood Illness in Developing Countries: A Systematic Review

**DOI:** 10.1371/journal.pone.0093427

**Published:** 2014-04-09

**Authors:** Pascal Geldsetzer, Thomas Christie Williams, Amir Kirolos, Sarah Mitchell, Louise Alison Ratcliffe, Maya Kate Kohli-Lynch, Esther Jill Laura Bischoff, Sophie Cameron, Harry Campbell

**Affiliations:** 1 Department of Global Health & Population, Harvard School of Public Health, Boston, Massachusetts, United States of America; 2 Public Health Sciences Section, Division of Community Health Sciences, University of Edinburgh, Edinburgh, United Kingdom; University of Liverpool, United Kingdom

## Abstract

**Background:**

Pneumonia, diarrhoea, and malaria are among the leading causes of death in children. These deaths are largely preventable if appropriate care is sought early. This review aimed to determine the percentage of caregivers in low- and middle-income countries (LMICs) with a child less than 5 years who were able to recognise illness in their child and subsequently sought care from different types of healthcare providers.

**Methods and Findings:**

We conducted a systematic literature review of studies that reported recognition of, and/or care seeking for episodes of diarrhoea, pneumonia or malaria in LMICs. The review is registered with PROSPERO (registration number: CRD42011001654). Ninety-one studies met the inclusion criteria. Eighteen studies reported data on caregiver recognition of disease and seventy-seven studies on care seeking. The median sensitivity of recognition of diarrhoea, malaria and pneumonia was low (36.0%, 37.4%, and 45.8%, respectively). A median of 73.0% of caregivers sought care outside the home. Care seeking from community health workers (median: 5.4% for diarrhoea, 4.2% for pneumonia, and 1.3% for malaria) and the use of oral rehydration therapy (median: 34%) was low.

**Conclusions:**

Given the importance of this topic to child survival programmes there are few published studies. Recognition of diarrhoea, malaria and pneumonia by caregivers is generally poor and represents a key factor to address in attempts to improve health care utilisation. In addition, considering that oral rehydration therapy has been widely recommended for over forty years, its use remains disappointingly low. Similarly, the reported levels of care seeking from community health workers in the included studies are low even though global action plans to address these illnesses promote community case management. Giving greater priority to research on care seeking could provide crucial evidence to inform child mortality programmes.

## Introduction

In 2012 it was estimated that 6.6 million children under the age of five died worldwide [1], eighty-two per cent of which in Sub-Saharan Africa and Southern Asia. Thirty-three per cent of these deaths were due to pneumonia (17%), diarrhoea (9%) and malaria (7%).

Morbidity and mortality from these diseases can be reduced considerably if care is sought early. Thus, the ability of caregivers to recognise and seek appropriate care for these common childhood illnesses is instrumental in reducing child deaths in low-and middle-income countries (LMICs) and in reaching the Millennium Development Goal 4 target of reducing child mortality by two thirds by 2015 [2].

Recognising the importance of care seeking, the World Health Organisation (WHO) and UNICEF highlighted activities to improve family and community health practices (including disease recognition and care seeking) as one of the three central components of the Integrated Management of Childhood Illness (IMCI) strategy [3]. The IMCI strategy has been introduced in the majority of LMICs with moderate to high child mortality and has been shown to be effective in a number of settings [4].

The importance of caregivers’ ability to recognise and seek appropriate care for their children is also one of the recommended key activities in the WHO’s and UNICEF’s Global Action Plan for the Control of Pneumonia and Diarrhoea [5]. In addition, a research priority setting exercise conducted by WHO using the Child Health and Nutrition Research Initiative (CHNRI) methodology identified the investigation of barriers to healthcare seeking and healthcare access as the highest primary research priority for reducing mortality from childhood pneumonia worldwide [6].

Herbert et al. have recently performed a systematic review of the percentage of caregivers who seek care for neonatal illness from different categories of healthcare providers [7]. However, there has not been a review of the literature on the recognition of, and care seeking behaviour for, the three main infectious causes of childhood mortality worldwide: pneumonia, diarrhoea and malaria. This review aims to estimate the percentage of caregivers in LMICs with a child of less than 5 years who were able to recognise signs of pneumonia, diarrhoea and malaria in their child and subsequently sought care from different types of health care providers.

## Methods

### Search Strategy

We reviewed published data on childhood pneumonia, diarrhoeal disease and malaria over the period from 1966 to November 2012. Searches were conducted of the following electronic databases: Medline [8], Embase [9], WHOLIS [10], Web of Knowledge (previously Web of Science) [11], LILACs [12], PAHO [13] and IndMED [14]. The databases were searched using key words and medical subject headings (MESH) for: pneumonia, diarrhoea, malaria, caregiver, recognition, danger signs, and care seeking. The searches were limited to published literature in children less than 5 years with abstracts in English. Otherwise, no restrictions were placed on publication language or type. See Table S1 for a complete list of search terms, formatted for Ovid Medline and Embase, and Table S2 for the study’s PRISMA checklist. The review is registered with PROSPERO (registration number: CRD42012003216) [15].

The titles and abstracts of retrieved studies were reviewed for relevance using the inclusion criteria detailed below. The full-text versions of potentially relevant articles were then analysed according to the inclusion criteria. Reference lists of all included studies were checked for additional references [16]. All citations were imported into an electronic database (Mendeley).

### Inclusion/Exclusion Criteria

Studies were included if: 1) the study presented original data, 2) the study was carried out in a LMIC, 3) the sample size was ≥50 participants, and 4) the study provided data on illness-recognition or care seeking for children <5 years of age. In addition, for recognition of disease, the study had to present quantitative data on the sensitivity and specificity of caregivers’ ability to recognise a disease or certain clinical features of the disease in their child. For care seeking, the study had to present quantitative data on the percentage of caregivers who sought healthcare for their child.

We excluded qualitative studies that described determinants of care seeking rather than enumerating care seeking events. Some publications described care seeking for two or three disease entities. If care seeking data were given separately for each disease entity they were included in the review.

### Definitions

#### Recognition of illness

For malaria, caregivers’ abilities to recognise a fever was compared to a thermometer measurement of axillary temperature, and their ability to recognise pallor to a haemoglobin measurement or haematocrit. Caregivers’ ability to recognise malaria was compared to a diagnosis made with a positive blood film, with (2 studies) or without (1 study) a raised temperature.

For dehydration during diarrhoeal disease, the ability of the caregiver to recognise features of dehydration was compared to a diagnosis made by a physician. For diarrhoea, the caregiver’s ability to describe diarrhoea was compared with a diagnosis using specified criteria. In two studies this was the WHO diagnostic criteria of at least three loose or liquid stools in a 24 hour period, and one study used a definition of at least 3 stools in 12 hours of daylight.

For pneumonia, the ability of caregivers to recognise rapid breathing and chest indrawing was compared to an assessment made by the study researchers. In all cases the WHO definition of pneumonia based on rapid breathing was used. This defines rapid breathing as a rate >60 breaths per minute in a child aged <2 months, >50 breaths per minute in a child aged <1 year, and >40 breaths per minute in a child over 1 year of age. The data was analysed separately for studies that employed open questioning (caregivers were asked to describe features of their child’s illness) and closed questioning (caregivers were asked whether the child had rapid breathing or chest indrawing).

#### Care seeking

Care seeking was defined as any care sought outside the home for an unwell child with a suspected episode of diarrhoea, malaria or pneumonia. A caregiver was defined as the individual who sought, or would have sought care for a sick child. In most cases this was a family member, most frequently the mother. For pneumonia, three studies specified acute respiratory infections (ARI) rather than acute lower respiratory tract infections (ALRI) and did not offer further qualifications. The remainder used the term “severe ARI” or reported signs suggesting pneumonia.

The following definitions of healthcare providers were adopted: ‘all health facilities’ included all government and private healthcare providers, pharmacies and traditional healers; ‘appropriate providers’ included all government and trained private health practitioners, but not traditional healers, pharmacies and unqualified medical practitioners; ‘all government providers’ also included non-governmental organisations (NGOs), which provided a free service, and was further subdivided into hospitals, health centres/clinics, and community health workers; ‘private facility’ included private hospitals and private health centres and clinics; ‘pharmacy’ referred to public and private pharmacies, chemists and drug vendors; and ‘traditional healer’ referred to practitioners of alternative medicine. Caregivers sought ‘no treatment’ when they neither gave home care nor sought external care. Oral rehydration therapy (ORT) for diarrhoea included oral rehydration solution (ORS) and sugar salt solution (SSS) as well as treatment given at home and that prescribed by care providers. If figures were provided only for ORT given at home, and did not include that supplied by care providers, the data was excluded from the analysis.

### Data Extraction and Assessment of the Studies

Data were extracted from studies and entered into Microsoft Excel spreadsheets. For intervention studies, only data from the control arm of the study or baseline data were included in the analysis. In addition to the data for illness recognition and care seeking, the following variables were extracted for each study: study location and geographical setting, date of data collection, total number of participants in the study, number of participants with an illness or suspected illness, sampling method, study design (including prospective or retrospective data collection), recall period, and the study’s definition of the disease entity and of recognition of the disease and/or care seeking for the disease.

If the sensitivity, specificity, and positive and negative predictive value of caregiver recognition of malaria, diarrhoea, pneumonia or features of the diseases were given, these data were extracted. Otherwise they were calculated from the published data, where possible. For care seeking, the proportion of caregivers that sought care and where they sought care was tabulated. In some studies data were given for both hypothetical and actual health care seeking behaviour. In these studies data on actual care seeking behaviour was used. In cases where only data on hypothetical care seeking behaviour was reported, this data was used. When care seeking from multiple providers was reported, then the first or preferred choice was extracted. Where possible, we calculated the percentage of care seeking from ‘any health facility’ and ‘any appropriate health facility’ if they were not stated in the study. Due to the wide variation in study designs, a meta-analysis was not possible. Instead, the median and range were calculated for care seeking within each category.

## Results

### Results of the Literature Search

**Figure 1:**
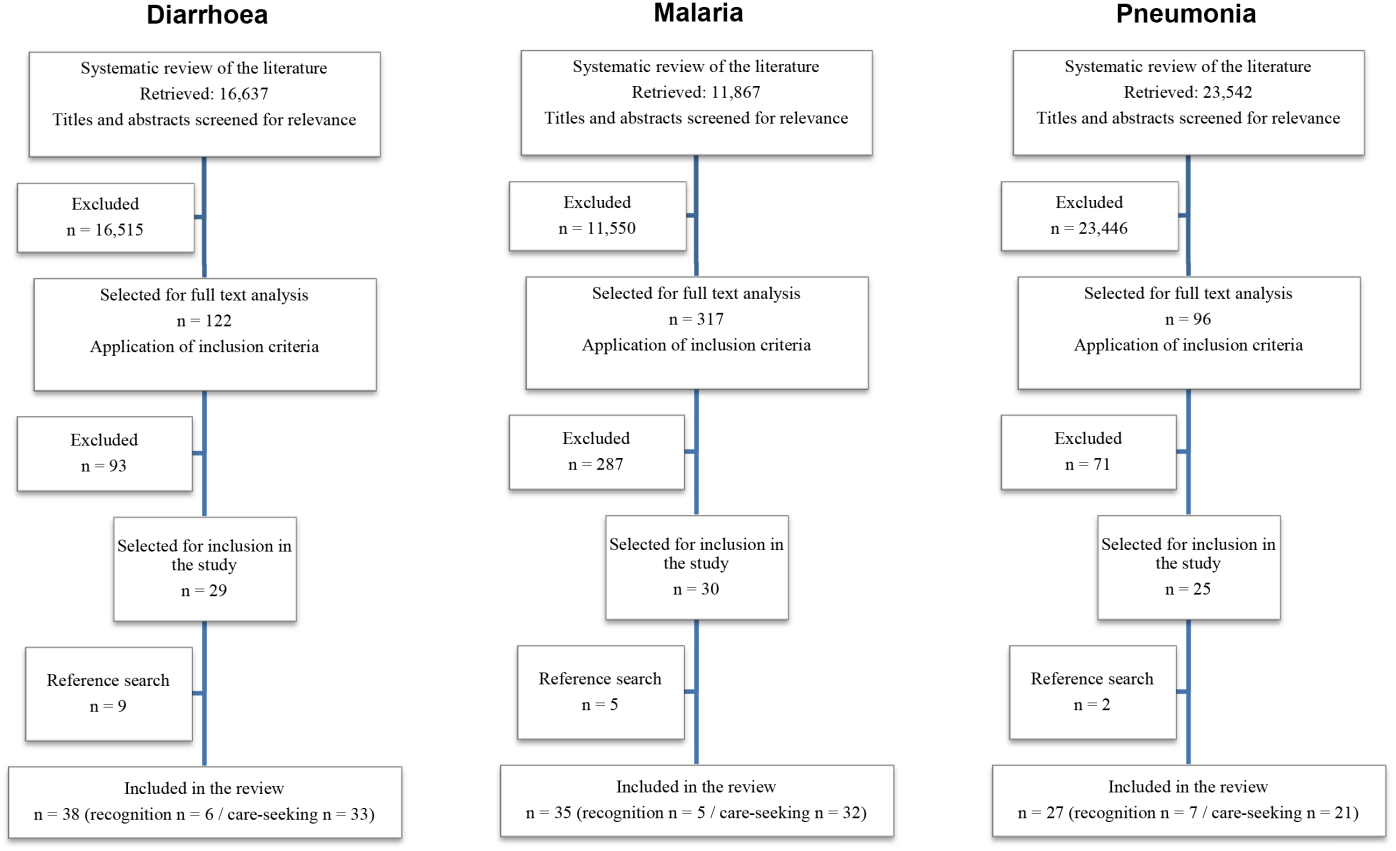
Summary of the literature searches.

The searches retrieved 16,637 studies for diarrhoea, 11,867 for malaria, and 23,542 for pneumonia, (Figure 1). After analysis of the full text, application of the inclusion criteria, and a reference search, 91 studies were included in the analysis (Table 1). 89 of these studies were in English; two were in French. 18 studies looked at recognition of disease, and 77 at care seeking behaviour (three studies contained data on both recognition of, and care seeking for the disease). Studies were excluded most commonly for being conducted in a high-income country, including children aged 5 years or older or not specifying the age of included children, having a sample size of less than 50 participants, collecting only qualitative data, and not presenting data for separate disease entities.

**Table 1:** Studies retrieved from literature search.

Illness category	Total number of articles retrieved	Selected for fulltext review	Included in the review^1^	With data on recognitionof illness	With data on care seeking for illness
Diarrhoea	16,637	122	38	6	33
Malaria	11,867	317	35	5	32
Pneumonia	23,542	96	27	7	21
Total			91^2^	18	77^2^

1 This number includes studies found through searching the references of already included studies.

2 Five studies examined two diseases, two studies examined all three diseases and three studies contained data on both recognition of disease and care seeking.

For each illness category, there were more studies with data on care seeking than for recognition of illness. The largest number of studies was found for diarrhoeal disease, and the fewest for pneumonia (38 and 27 studies respectively).

### Study Characteristics

The majority (75/91) of selected studies were carried out within a single country. One study presented data on care seeking for diarrhoea in 6 countries in Africa, Asia and Latin America [17] and one study on care seeking for malaria in 6 African countries [18]. The majority of the studies were carried out in Africa (13/18 for recognition of disease, 50/77 for care seeking, 60/91 in total) and Asia (5/18 for recognition of disease, 24/77 for care seeking, 29/91 in total). Few studies were found for Latin America (none for recognition of disease, 6/77 for care seeking).

For recognition of disease (Table 2), 10 of 18 studies were undertaken between 1990 and 1999. Seven studies reported recognition of features of pneumonia, six of diarrhoea, and five of malaria. For care seeking behaviour (Table 3), there was an increase in the number of studies published over time: 9 before 1990, 23 between 1990 and 1999, and 43 between 2000 and 2009. Most studies on care seeking behaviour (48/77) had fewer than 1000 participants; 11 had over 5000 participants. The majority of studies (71/77) examined care seeking for children under the age of 5 years. In terms of study design (Table 4), most studies were retrospective (54/77) and a significant proportion (17/77) examined hypothetical decisions made by caregivers rather than actual care seeking behaviour; these will be referred to as “simulated case studies”.

**Table 2:** Characteristics of studies on recognition of illness.

		Diarrhoea *(n = 6)*	Malaria *(n = 5)*	Pneumonia *(n = 7)*	Total *(n = 18)*
**Location**	Africa	4	5	4	13
	Asia	2	0	3	5
	Latin America	0	0	0	0
**Date of data collection** 1	1989 or earlier	4	0	1	5
	1990–1999	1	4	5	10
	2000–2009	0	1	1	2
	2010–2012	1	0	0	1
**Age of participants**	<1 years	0	0	2	2
	<2 years	2	1	0	3
	<3 years	3	0	0	3
	<5 years	1	4	5	10
**Sample size**	50–100	1	0	0	1
	101–500	3	3	4	10
	501–1000	0	1	1	2
	1001–5000	1	1	1	3
	>5000	1	0	1	2

1 This is the mid-year of data collection. If the date of data collection was not stated, year of publication was used instead.

**Table 3:** Characteristics of studies on care seeking behavior.

		Diarrhoea *(n = 33)*	Malaria *(n = 32)*	Pneumonia *(n = 21)*	Total^1^ *(n = 77)*
**Location**	Africa	17	31	8	49
	Asia	15	1	10	24
	Latin America	3	0	3	6
**Date of data collection**	Before 1990	6	3	1	9
	1990–1999	9	6	9	23
	2000–2009	17	21	11	42
	2010–2012	1	2	0	3
**Age of participants**	<2 years	0	1	0	1
	<3 years	2	2	1	5
	<5 years	31	29	20	71
**Sample size**	50–100	2	4	2	7
	101–500	7	12	9	28
	501–1000	5	6	2	13
	1001–5000	11	8	6	18
	>5000	8	2	2	11

1 Five studies examined two diseases, two studies examined all three diseases and one study was carried out in three continents (Africa, Asia and Latin America).

**Table 4:** Features of studies on care seeking behavior.

Study design			Definitions		Study setting
Prospective, retrospective orsimulated case study^1^		Recall period	Definition of disease	Definition of care seeking	
**Total n = 77**					
Prospective: 6	≤2 weeks: 33		Clinical criteria: 42	Seeking care outside the home: 18	Rural: 39
Retrospective: 54	>2 weeks: 16		Caregiver description: 26	Seeking care from defined providers: 59	Urban: 16
Simulated case study: 17	Not stated: 5		Not defined: 9		Rural/urban^2^: 19
	Not retrospective: 23				Not specified: 3
**Diarrhoea n = 33**					
Prospective: 3	≤2 weeks: 21		Clinical criteria: 20	Seeking care outside the home: 17	Rural: 12
Retrospective: 27 Simulated casestudy: 3	>2 weeks: 5		Caregiver description: 4	Seeking care from defined providers: 16	Urban: 11
	Not stated: 1		Not defined: 9		Rural/urban^2^: 9
	Not retrospective: 6				Not specified: 1
**Malaria n = 32**					
Prospective: 3	≤2 weeks: 14		Clinical criteria: 9	Seeking care outside the home: 4	Rural: 17
Retrospective: 23	>2 weeks: 7		Caregiver description: 22	Seeking care from defined providers: 28	Urban: 4
Simulated case study: 6	Not stated: 2		Not defined: 1		Rural/urban^2^: 9
	Not retrospective: 9				Not specified: 2
**Pneumonia n = 21**					
Prospective: 0	≤2 weeks: 7		Clinical criteria: 19	Seeking care outside the home: 3	Rural: 15
Retrospective: 13	>2 weeks: 4		Caregiver description: 2	Seeking care from defined providers: 18	Urban: 5
Simulated case study: 8	Not stated: 2		Not defined: 0		Rural/urban^2^: 1
	Not retrospective: 8				Not specified: 0

1 Simulated case studies presented caregivers with a scenario of an illness in their child and asked whether and where he/she would seek care.

2 Rural/urban refers to studies that were carried out in both urban and rural areas.

42/77 studies on care seeking used clinical criteria to define diarrhoea, malaria or pneumonia, 26/77 used caregiver descriptions of illness, and 9/77 did not give any definition of disease. Most studies (59/77) defined care seeking as seeking care from defined health care providers; the remainder defined it as seeking care outside the home. Approximately half of the studies were undertaken in rural locations (40/77).

### Recognition of Childhood Illness

Definitions of disease varied between studies, as described in the methods. Three studies reported sensitivities and specificities for the overall caregiver recognition of malaria and diarrhoea. Only one study [19] looked at caregiver recognition of pneumonia, while six studies looked at recognition of rapid breathing and chest indrawing (Table 5). Median sensitivities were 37.4% for the recognition of malaria, 36.0% for diarrhoea, and 45.8% for pneumonia. The specificity of caregiver recognition of disease was highest for diarrhoea (96.0%) and lowest for malaria (58.0%); the specificity for recognition of pneumonia was 67.1%. The positive predictive value of a caregiver diagnosis of illness was highest for diarrhoea (67.7%) and lowest for pneumonia (28.9%).

**Table 5:** Caregivers’ recognition of diarrhoea, malaria, pneumonia and clinical features of these diseases.

		Median % and range			
Condition	Feature recognized by caregivers	Sensitivity	Specificity	Positive predictive value	Negative predictive value
***Diarrhoea***	Diarrhoea [30], [84], [85]	36.0 (14.3–54.6)	96.0 (94.7–97.1)	67.7 (61.1–70.6)	86.6 (55.4–96.3)
	Thirst [86]–[88]	85.0 (73.0–89.0)	29.0 (10.0–30.0)	-	-
	Dry mouth [86]–[88]	71.0 (67.0–74.0)	35.0 (30.0–44.0)	-	-
	Weakness [86]–[88]	88.0 (67.0–95.0)	20.0 (13.0–50.0)	-	-
	Sunken eyes [86]–[88]	74.0 (64.0–94.0)	49.0 (36.0–60.0)	-	-
	Decreased urine production [86]–[88]	21.0 (11.0–35.0)	80.0 (70.0–87.0)	-	-
	Sunken fontanelle [87], [88]	75.5 (71.0–80.0)	24.5 (20.0–29.0)	-	-
	No tears [86]	16.0	80.0	-	-
***Malaria***	Malaria [52], [89], [90]	37.4 (32.0–40.0)	58.0 (55.0–92.0)	36.8 (13.8–61.2)	83.6 (34.3–90.2)
	Anaemia^1^ [89], [91]	22.4 (4.1–46.8)	81.5 (95.6–67.5)	15.9 (10.4–21.4)	86.2 (78.5–94.0)
	Fever^2^ [91], [92]	64.7 (53.3–76.2)	87.1 (80.4–87.1)	45.1	90.7 (85.1–96.4)
***Pneumonia***	Moderate pneumonia^3^ [19]	45.8	67.1	28.9	80.9
	Rapid breathing (open questioning^4^)[31], [93]–[95]	22.0 (15.6–78.0)	97.6 (84.0–100.0)	100.0	37.2
	Rapid breathing (closed questioning^5^)[93], [95]–[97]	69.8 (48.7–78.0)	71.5 (34.0–90.0)	77.0 (73.1–93.2)	59.0 (52.0–61.6)
	Recognition of chest indrawing (open questioning) [31], [93], [95]	17.0 (7.5–35.3)	97.2 (93.0–100.0)	100.0	41.9
	Recognition of chest indrawing (closed questioning) [93], [95], [97]	68.0 (56.7–69.0)	64.0 (49.0–100.0)	89.5 (79.0–100.0)	56.8 (53.0–60.6)

1 Defined as a haemoglobin concentration of<7 gram per decilitre or haematocrit <25%.

2 Defined as an axillary temperature ≥37.5°C.

3 Defined as the presence of a cough and a raised respiratory rate.

4 Open questioning: the caregiver was asked to describe features of the child’s illness.

5 Closed questioning: the caregiver was asked whether the child suffered from rapid breathing or chest indrawing.

The median sensitivity of recognition of anaemia by caregivers was low (22.4%), but the sensitivity of recognition of fever was higher (64.7%). Caregivers’ sensitivity of detection of rapid breathing was markedly increased on closed compared to open questioning (69.8% vs 22.0%), as was recognition of chest indrawing (68.0% vs 17.0%). However closed questioning led to a fall in the specificity (97.6% vs. 71.5% for rapid breathing, 97.2% vs. 64.0% for chest indrawing). The sensitivity of caregiver recognition of features of moderate to severe dehydration was high: 88.0% for weakness, 85.0% for thirst, 75.5% for a sunken fontanelle, 74.0% for sunken eyes, and 71.0% for dry mouth. Caregivers were less able to recognise decreased urine production (sensitivity: 21.0%) or the absence of tears (sensitivity: 16.0%).

### Care Seeking for Childhood Illness

In 2012, the countries with the highest number of child deaths, in descending order, were India, Nigeria, the Democratic Republic of the Congo (DRC), Pakistan and China [1]. Table 6 shows the number of studies on care seeking that were carried out in each of these five countries. 6 and 11 studies were carried out in India and Nigeria, respectively. However, only 2 of the included studies were carried out in each the DRC, Pakistan and China. We did not identify any studies from Nigeria, China or the DRC that investigated care seeking for pneumonia. Our literature search may however have missed articles in Chinese or French without an English abstract.

**Table 6:** Number of studies on care seeking behaviour, by the 5 countries with the highest number of under-five deaths^1^.

Country	Number of studies
India [20], [27], [42], [98]–[100]	6
Nigeria[17], [18], [25], [34], [36], [58], [59], [62], [101]–[103]	11
Democratic Republic of the Congo [17], [18]	2
Pakistan [48], [53]	2
China [28], [104]	2

1 The five countries with the highest number of under-five deaths were taken from the annual report by the UN Interagency Group for Child Mortality Estimation [1].

Table 7 summarises the studies on care seeking behaviour for all three diseases for all continents. More detailed data for each continent is provided in Table 8 (Africa), Table 9 (Asia) and Table 10 (Latin America). Table 11 shows the same data separated by each disease entity.

**Table 7:** The median percentage of caregivers who sought healthcare, by disease (all continents)^1^.

		Appropriate health facilities									
			Government facilities								
	All health facilities	All appropriate facilities^2^	Gov. Hospital^3^	Health centre or clinic	CHW	All gov. facilities	Private facilities	Pharmacy or drug vendor	Trad. healer	Home care	No care
**Diarrhoea** [17], [20]–[23], [27]–[30], [36]–[41], [46]–[48], [54], [59]–[66], [69], [98], [104]–[107]	68.5 (14.9–96.0)	44.5 (7.3–87.0)	8.0 (2.0–18.0)	21.2(0.3–80.0)	5.4(5.0–21.0)	30.8 (7.3–85.0)	22.0 (1.0–68.8)	16.7 (4.9–55.7)	6.3 (1.4–20.7)	32.1 (4.0–68.0) ORT: 34.0 (13.4–68.1)	21.3 (6.0–39.4)
**Malaria** [18], [21], [22], [24]–[26], [32]–[34], [44], [45], [49], [50], [52], [58], [68], [89], [100]–[103], [105], [107]–[116]	72.6 (6.1–100.0)	42.4 (6.1–98.4)	9.1 (1.2–20.0)	21.0(5.5–71.0)	1.3(0–40.2)	21.3 (10.8–81.5)	7.2(1.9–28.6)	30.8 (1.6–49.7)	3.5 (1.2–60.0)	38.7 (4.5–88.0)	8.1 (0.0–51.7)
**Pneumonia** [4], [19]–[22], [29], [38], [42], [43], [51], [53], [55]–[57], [79], [99], [117]–[121]	91.3 (5.3–100)	84.0 (20.9–100.0)	46.0(19.6–60.0)	32.8(18.0–56.3)	4.2(2.0–18.0)	50.0(20.9–100.0)	24.0(6.7–49.9)	15.2(2.5–27.6)	21.4 (3.1–64.4)	20.0 (2.8–94.6)	11.0 (0.9–11.5)

1 The range is shown in brackets if there are two or more data points. The definition of each category of healthcare is stated in the methods. The values do not add up to 100% because of the varying categorisation of healthcare providers in the included studies and because some studies recorded more than one care seeking event.

2 Appropriate health facilities included all government and trained private health practitioners, but not traditional healers, pharmacies and unqualified medical practitioners.

3 Abbreviations: Gov.  =  Governmental; CHW  =  Community Health Worker; Trad. Healer = Traditional Healer; ORT  =  Oral Rehydration Therapy.

**Table 8:** The median percentage of caregivers who sought healthcare, by disease (Africa)^1^.

		Appropriate health facilities									
			Government facilities								
	All health facilities	All appropriate facilities^2^	Gov. Hospital^3^	Health centre or clinic	CHW	All gov. facilities	Private facilities	Pharmacy or drug vendor	Trad. healer	Home care	No care
**Diarrhoea** [17], [21], [22], [29], [30], [36], [37], [41], [46], [47], [60], [62], [63], [65], [66], [105], [107]	67.3 (14.9–86.0)	39.0 (7.3–68.9)	8.8 (5.3–17.4)	28.0 (2.0–42.5)	5.4 (5.0–21.0)	18.7 (7.3–66.2)	11.7 (1.0–37.6)	22.2 (10.5–45.0)	7.0 (2.2–20.7)	32.4 (14.0–60.7) ORT: 40.5 (13.4–68.1)	30.8 (6.0–39.4)
**Malaria** [18], [22], [23], [25]–[27], [33]–[35], [45], [46], [50], [51],[53], [59], [69], [91], [102]–[104], [106], [108]–[117]	72.1 (6.1–100.0)	41.6 (6.1–98.4)	9.1 (1.2–20.0)	21.0 (5.5–71.0)	1.3 (0–40.2)	29.5 (10.8–72.3)	7.2 (1.9–28.6)	30.8 (1.6–49.7)	3.5 (1.2–60.0)	38.7 (4.5–88.0)	8.1 (0.0–51.7)
**Pneumonia** [22], [23], [30], [44], [52], [119]–[121]	98.0 (62.9–100.0)	86.0 (27.2–100.0)	46.0 (19.6–51.5)	35.6 (20.0–56.3)	3.1 (2.0–4.2)	57.0 (35.6–100.0)	20.0 (6.7–24.0)	16.6 (2.5–27.6)	21.4 (3.1–64.4)	-	0.0

1 The range is shown in brackets if there are two or more data points. The definition of each category of healthcare is stated in the methods. The values do not add up to 100% because of the varying categorisation of healthcare providers in the included studies and because some studies recorded more than one care seeking event.

2 Appropriate health facilities included all government and trained private health practitioners, but not traditional healers, pharmacies and unqualified medical practitioners.

3 Abbreviations: Gov  =  Governmental; CHW  =  Community Health Worker; Trad. Healer = Traditional Healer; ORT  =  Oral Rehydration Therapy; -  =  no data available.

**Table 9:** The median percentage of caregivers who sought healthcare, by disease (Asia)^1^.

		Appropriate health facilities									
			Government facilities								
	All health facilities	All appropriate facilities^2^	Gov. Hospital^3^	Health centre or clinic	CHW	All gov. facilities	Privatefacilities	Pharmacy or drug vendor	Trad. healer	Home care	No care
**Diarrhoea** [17], [21], [28], [29], [39], [41], [49], [55], [60], [62], [65], [70], [99], [105], [107]	78.7 (32.0–96.0)	65.0 (25.4–87.0)	7.1 (2.0–18.0)	25.1 (0.3–80.0)	-	32.2 (15.0–85.0)	29.3 (5.5–68.8)	10.5 (4.0–55.7)	3.0 (1.4–5.0)	19.8 (4.0–68.0) ORT: 34.0 (29.0–46.0)	20.0 (8.75–21.3)
**Malaria** [101]	81.5	81.5	-	-	1.2	81.5	-	-	-	-	-
**Pneumonia** [4], [19], [21], [39], [43], [54], [57], [80], [100], [122]	80.0 (5.3–92.5)	68.8 (20.9–92.5)	60.0	32.5 (18.0–47.0)	-	33.5 (20.9–60.0)	49.0	-	-	25.6 (2.8–94.6)	6.2 (0.9–11.5)

1 The range is shown in brackets if there are two or more data points. The definition of each category of healthcare is stated in the methods. The values do not add up to 100% because of the varying categorisation of healthcare providers in the included studies and because some studies recorded more than one care seeking event.

2 Appropriate health facilities included all government and trained private health practitioners, but not traditional healers, pharmacies and unqualified medical practitioners.

3 Abbreviations: Gov  =  Governmental; CHW  =  Community Health Worker; Trad. Healer = Traditional Healer; ORT  =  Oral Rehydration Therapy; -  =  no data available.

**Table 10:** The median percentage of caregivers who sought healthcare, by disease (Latin America)^1^.

		Appropriate health facilities									
			Government facilities								
	All health facilities	All appropriate facilities^2^	Gov. Hospital^3^	Health centre or clinic	CHW	All gov. facilities	Privatefacilities	Pharmacy or drug vendor	Trad.healer	Home care	No care
**Diarrhoea** [17], [24], [40]	40.5 (22.0–59.0)	46.0 (33.0–59.0)	8.0	10.0	-	18.0	-	-	-	41.0 ORT: 23.2	20.0
**Malaria**	-	-	-	-	-	-	-	-	-	-	-
**Pneumonia** [56], [58], [118]	87.5 (83.5–91.6)	89.0 (83.5–94.6)	30.9	30.0	-	60.9	46.6	13.4 (11.6–15.2)	3.3	5.5	11.0

1 The range is shown in brackets if there are two or more data points. The definition of each category of healthcare is stated in the methods. The values do not add up to 100% because of the varying categorisation of healthcare providers in the included studies and because some studies recorded more than one care seeking event.

2 Appropriate health facilities included all government and trained private health practitioners, but not traditional healers, pharmacies and unqualified medical practitioners.

3 Abbreviations: Gov  =  Governmental; CHW  =  Community Health Worker; Trad. Healer = Traditional Healer; ORT  =  Oral Rehydration Therapy; -  =  no data available.

**Table 11:** The median percentage of caregivers who sought healthcare, by disease and continent^1^.

		Appropriate health facilities									
			Government facilities								
	All health facilities	All appropriate facilities^2^	Gov. Hospital^3^	Health centre or clinic	CHW	All gov. facilities	Private facilities	Pharmacy or drug vendor	Trad. healer	Home care	No care
***Diarrhoea***											
**Africa** [17], [22], [23], [30], [31], [37], [38], [42], [47], [48], [61], [63], [64], [66], [67], [106], [108]	67.3 (14.9–86.0)	39.0 (7.3–68.9)	8.8 (5.3–17.4)	28.0 (2.0–42.5)	5.4 (5.0–21.0)	18.7 (7.3–66.2)	11.7 (1.0–37.6)	22.2 (10.5–45.0)	7.0 (2.2–20.7)	32.4 (14.0–60.7) ORT: 40.5 (13.4–68.1)	30.8 (6.0–39.4)
**Asia** [17], [21], [28], [29], [39], [41], [49], [55], [60], [62], [65], [70], [99], [105], [107]	78.7 (32.0–96.0)	65.0 (25.4–87.0)	7.1 (2.0–18.0)	25.1 (0.3–80.0)	-	32.2 (15.0–85.0)	29.3 (5.5–68.8)	10.5 (4.0–55.7)	3.0 (1.4–5.0)	19.8 (4.0–68.0) ORT: 34.0 (29.0–46.0)	20.0 (8.75–21.3)
**Latin America** [17], [24], [40]	40.5 (22.0–59.0)	46.0 (33.0–59.0)	8.0	10.0	-	18.0	-	-	-	41.0 ORT: 23.2	20.0
***Pneumonia***											
**Africa** [22], [23], [30], [44], [52], [119]–[121]	98.0 (62.9–100.0)	86.0 (27.2–100.0)	46.0 (19.6–51.5)	35.6 (20.0–56.3)	3.1 (2.0–4.2)	57.0 (35.6–100.0)	20.0 (6.7–24.0)	16.6 (2.5–27.6)	21.4 (3.1–64.4)	-	0.0
**Asia** [4], [19], [21], [39], [43], [54], [57], [80], [100], [122]	80.0 (5.3–92.5)	68.8 (20.9–92.5)	60.0	32.5 (18.0–47.0)	-	33.5 (20.9–60.0)	49.0	-	-	25.6 (2.8–94.6)	6.2 (0.9–11.5)
**Latin America** [56], [58], [118]	87.5 (83.5–91.6)	89.0 (83.5–94.6)	30.9	30.0	-	60.9	46.6	13.4 (11.6–15.2)	3.3	5.5	11.0
***Malaria***											
**Africa** [18], [22], [23], [25]–[27], [33]–[35], [45], [46], [50], [51], [53], [59], [69], [91], [102]–[104], [106], [108]–[117]	72.1 (6.1–100.0)	41.6 (6.1–98.4)	9.1 (1.2–20.0)	21.0 (5.5–71.0)	1.3 (0.0–40.2)	29.5 (10.8–72.3)	7.25 (1.9–28.6)	30.8 (1.6–49.7)	3.5 (1.2–60.0)	38.7 (4.5–88.0)	8.1 (0.0–51.7)
**Asia** [101]	81.5	81.5	-	-	1.2	81.5	-	-	-	-	-
**Latin America**	-	-	-	-	-	-	-	-	-	-	-

1 The range is shown in brackets if there are two or more data points. The definition of each category of healthcare is stated in the methods. The values do not add up to 100% because of the varying categorisation of healthcare providers in the included studies and because some studies recorded more than one care seeking event.

2 Appropriate health facilities included all government and trained private health practitioners, but not traditional healers, pharmacies and unqualified medical practitioners.

3 Abbreviations: Gov  =  Governmental; CHW  =  Community Health Worker; Trad. Healer = Traditional Healer; ORT  =  Oral Rehydration Therapy; -  =  no data available.

A median of 73.0% (range: 5.3%–100.0%) of caregivers sought care from a healthcare provider when their child was suffering from diarrhoea, malaria or pneumonia and a median of 44.9% (range: 6.1%–100.0%) sought care from appropriate providers. Care seeking was highest for pneumonia with a median of 91.3% of caregivers seeking care from any provider, and lowest for diarrhoea with 68.5% (Table 7). Seeking no care was commonest for diarrhoea (21.3%) and least common for malaria (8.1%). Appropriate care was sought most frequently for pneumonia (84.0%), and least frequently for malaria (42.5%). Government facilities were attended more frequently than private facilities for all diseases, except for pneumonia in Asia (only one study [20] provided data for care seeking from private care providers for pneumonia in Asia). Care seeking from community health workers (CHWs) was low: 5.4% for diarrhoea, 4.2% for pneumonia, and 1.3% for malaria. Caregivers sought care from pharmacies and drug vendors most frequently in cases of malaria (30.8%), and least frequently for pneumonia (15.2%). Traditional healers were consulted most frequently for pneumonia (21.4%). ORT was used by caregivers in 34.0% (range: 13.4%–68.1%) of cases of diarrhoea.

It was difficult to compare care seeking in Latin America to Asia or Africa, as only 6 studies were carried out in Latin America. Comparing Africa and Asia (see Table 11), carers were less likely to seek care for diarrhoea in Africa (67.3%) than in Asia (78.7%) but more likely to seek care for pneumonia (98.0% versus 80.0%). Care seeking from private care providers was more common in Asia than in Africa for both diarrhoea (29.3% versus 11.7%) and pneumonia (49.0% versus 20.0%). In both continents, carers were more likely to give no treatment for diarrhoea than for pneumonia. Rates of ORT use were low both in Africa (40.5%) and Asia (34.0%). For pneumonia and malaria, none of the included studies reported data on care seeking from pharmacies and traditional healers in Asia.

### Factors Influencing Care Seeking for Childhood Illness

Identifying studies which contained data on factors that influence care seeking was not a primary aim of this review. However, many of the studies included in the review presented data on influencing factors. This section summarises these studies’ findings.

#### Geography

18 studies examined the association between geographical factors and care seeking behaviour. Six studies [21]–[26] found that caregivers in urban areas were more likely to seek care than those in rural locations, and eight studies [26]–[33] showed that rates of care seeking were inversely related to the distance to the closest health facility. In addition, two studies [30], [34] showed that caregivers were more likely to seek traditional care if they were further away from a health facility.

#### Severity of illness

Twelve studies examined the association between illness severity and care seeking behaviour. Six studies [35]–[39] found that the more severe caregivers perceived the child’s illness to be, the more likely they were to seek care. The same association was present when severity of illness was defined by clinical criteria [37]–[42]. Of studies reporting data on fatal illness, a study on care seeking for pneumonia [43] found that rates of care seeking from appropriate providers had been high in children who died from the illness (100%). However, another two studies [44], [45] on fatal illness showed that care was sought initially from an appropriate provider in only 49.0% and 44.7% of cases, respectively.

#### Socioeconomic status and cost of health care

Eighteen studies examined links between socioeconomic status, the cost of care, and care seeking behaviour. Three studies [38], [46], [47] found no association between socioeconomic status and rates of care seeking behaviour. Four studies [22], [48]–[50] found a positive correlation between socioeconomic status and rates of care seeking. One study [28] reported a positive association between the level of maternal education and appropriate care seeking. Participants in six studies [21], [27], [33], [44], [51], [52] cited cost as a reason for not seeking care for children with illness. However, two studies [29], [53] commented on the low rates of appropriate care seeking despite health care being free of charge at the point of care in the study locations. One study [54] found that wealthier caregivers were more likely to attend hospitals than health centres when their child was ill, and another [55] reported that they were more likely to seek care from private providers.

#### Gender

Seven studies examined whether the gender of the child influenced care seeking. Four studies in Kenya, Ethiopia, Pakistan and Sri Lanka [21], [38], [50], [53] found no significant difference in care seeking rates between male and female children. Two studies carried out in Indonesia and Burkina Faso [30], [54], however, found that carers were more likely to seek care for boys than girls. One study looking at cases of fatal malaria [45] showed that carers were more likely to take boys to traditional healers than girls.

Two studies commented that attending a healthcare provider had frequently been delayed because mothers had wanted to consult the head of the household before seeking care [55], [56].

## Discussion

Treatments for childhood diarrhoea, malaria and pneumonia are usually very effective if care is sought in time. The challenge, therefore, is to implement on-going programmes, which educate caregivers to recognise when to seek care and which facilitate appropriate care seeking behaviour.

### Caregiver Recognition of Illness and Importance of Local Context

We found a paucity of studies with data on the sensitivity and specificity of caregiver recognition of disease, and furthermore a decrease in the number of these studies over time. The studies that were available showed that caregivers’ sensitivity for recognition of diarrhoea, malaria and pneumonia was low, with median sensitivities of 26.0%, 37.4% and 45.8% respectively. The positive predictive value of a caregiver diagnosis of an illness was highest for diarrhoea (67.7%) and lowest for pneumonia (28.9%). Definitions of disease varied between the studies, making comparison difficult. Furthermore, the sensitivity and specificity of caregiver recognition of disease depended on how caregivers were questioned. For pneumonia, for example, asking closed rather than open questions led to a significant increase in sensitivity but a corresponding decrease in specificity.

More high-quality studies on the recognition of childhood illness are needed to allow for the design of health education and communication strategies that are informed by what diseases and clinical features caregivers already recognise and which ones they do not. Several of the included studies suggest that health education programmes can improve caregiver recognition of childhood illness and appropriate care seeking. A cross-sectional study in Peru [57] found that mothers who reported to have been exposed to a national pneumonia educational campaign on television were more likely to select rapid breathing and chest retraction as signs of pneumonia in a questionnaire. In addition, a before-and-after evaluation [58] of community information activities and a training of trainers for leaders of women groups in Nigeria found significant improvements in knowledge of the symptoms of malaria and its treatment, increased rates of appropriate care seeking for severely ill children, and reductions in rates of consultations with traditional healers for severely ill children (from 60% to 6.7% of cases). In addition, the IMCI strategy can be effective in improving appropriate care seeking. A cluster randomised trial of IMCI in Bangladesh [4] demonstrated the effectiveness of the facility-based component of IMCI in improving appropriate care-seeking for childhood illness. In this study, rates of appropriate care seeking for childhood illness were 20% in IMCI areas compared to 8% in control areas after three years. The subsequent employment of village health workers in IMCI areas further increased appropriate care seeking to 24% after 1.5 years compared to 5% in control areas.

Whilst education programmes can be effective, they must also be maintained over time. The included studies with data on the use of ORT for diarrhoea showed a fairly constant level of ORT use over time: a median of 31.0% (23.2%–46.0%) before 1990, 34.8% (15.7%–55.1%) from 1990 to 1999, and 36.3% (13.4%–68.1%) from 2000 to 2010 [20]–[23], [37], [48], [59]–[66]. The authors of one of the studies included in this review [66] commented that there had been no recent campaigns to encourage ORT use in the study areas in Kenya. They suggested that the previous generation of caregivers may in fact be more informed than the current one about the benefit of ORT use in diarrhoeal disease. Overall, the levels of ORT use (a median of 34.0% across all studies) are disappointingly low given that ORT was introduced and then recommended by the WHO more than 40 years ago, and that programmes have been promoting ORT for many years.

An evaluation of the IMCI strategy noted that global strategies must be accompanied by country level implementation guidelines [67]. Knowledge of local context is important to understand some of the factors that influence care seeking behaviour. We found that in most studies there was an inverse association between distance to a health care facility and the likelihood of seeking care. However, associations with other factors (such as socioeconomic status and gender) were more culturally or geographically dependent with results varying across studies. Despite cost being cited as a reason not to seek care by participants in some studies, several studies found a low uptake of government services even though these were free at the point of access [29], [40], [53], [55], [68], [69].

### Use of Data on Childhood Illness Reported by Caregivers in Disease Burden Estimations

In general, the sensitivity and specificity of caregiver recognition of malaria, pneumonia and diarrhoea was low (medians of 36% sensitivity and 96% specificity for diarrhoea; 46% sensitivity and 67% specificity for pneumonia; 37% sensitivity and 58% specificity for malaria). This strongly suggests that survey data based on these reports should not be used for disease burden estimations. It further suggests that reported diarrhoeal illness (but not pneumonia or malarial illness in a child) is of sufficiently high specificity to be a reasonably valid endpoint for evaluation of an intervention in a controlled trial.

### Low Utilisation of Community-based Health Workers

A recent review has highlighted the effectiveness of community-based interventions and platforms in improving breastfeeding rates, managing neonatal infections and childhood pneumonia and improving levels of care seeking [70], [71]. Community-based packaged interventions delivered through CHWs have been shown to improve immunization uptake and levels of care seeking for childhood illnesses [70], [72]–[74] and reduce levels of inappropriate use of antibiotics for diarrhoea and mortality and treatment failure rates for pneumonia [70], [71]. In this review we found very low levels of care seeking from CHWs, with a median of only 1.3% of caregivers utilising them for malaria, 4.2% for pneumonia, and 5.4% for diarrhoea. This low level of utilisation of CHWs is consistent with the recent multi-country IMCI evaluation, which reported that implementation of the community arm of IMCI has been disappointing and that interventions to improve care seeking had not been introduced [7]. This is of particular concern as community case management is now a major UNICEF and WHO strategy. Reasons given by caregivers for not using CHWs included perceived low status within the communities they worked in [60] and a lack of supplies and medications [33]. However, most of the included studies did not elaborate on reasons for low uptake of their services and reasons may include supply-side as well as demand-side factors.

### Private Practitioners

Several of the included studies found a fairly high rate of consultation with private providers in the presence of free governmental care [18], [53], [55], [69]. Caregivers were more likely to seek care from private facilities in Asia than Africa. This is perhaps not surprising, given that the proportion of private healthcare providers is generally larger in Asia [75]. Participants gave a variety of reasons for seeking care from private rather than public providers. These included convenience (both in terms of geography and opening times), prompt care and more courteous service. These findings are supported by a recent review, which found that the public sector appeared frequently to lack timeliness and hospitality towards patients [76]. We found in this review that participants in some studies stated they felt they obtained higher quality care from the private sector, and there was an increased likelihood of receiving what they perceived to be appropriate treatment (e.g. injections for pneumonia and antibiotics or anti-motility agents for diarrhoea).

Given the high proportion of people in LMICs seeking care from private providers, it has been suggested that one way to improve health outcomes is to invest in the private health care sector [77], [78] although recognising that there are significant challenges [77]. Indeed, three studies [18], [38], [53] in our review reported lower standards of care in the private sector.

### Traditional Healers and Pharmacies

Another source from which caregivers sought help were traditional healers. They were consulted most frequently in Africa for pneumonia, with a median of 20.8% seeking care. Reasons given for consulting traditional healers included cost [44], ease of access [30], [34], and perceived severity [60] or mildness of disease [29]. In general, they were associated with poorer health outcomes: seeking care from traditional healers in one study was associated with higher mortality [60] whilst another study found high rates of consultation with traditional healers among children who died [79]. Consultations with traditional healers were found in one study to result in delayed presentation to appropriate providers [56] and this is the most likely reason for poorer health outcomes.

This review also found high rates of care seeking from pharmacies and drug vendors. The highest rates (median: 30.8%) were for malaria in Africa. Given that rates of care seeking from pharmacies, drug vendors and traditional healers are high, the authors of several studies advocated working with these providers to increase uptake of appropriate treatments for diarrhoea [66], [80], malaria [44] and pneumonia [56]. A recent cluster-randomised controlled trial shows that such an approach can be successful. It evaluated the delivery of subsidised artemisinin-based combination therapy (ACT) to private retailers and found an increase of 40.2% in the number of children who received ACT on the day of fever or the following day in the intervention group compared to an increase of 14.6% in the control group (p<0.0001) [81].

The studies in this review demonstrate the importance of private care providers, pharmacies and traditional healers, in addition to government facilities, in providing care to children with diarrhoea, pneumonia and malaria. The studies also highlight the low uptake of care provided by community health workers and persistently low rates of ORT use. On the whole, there are few published studies on this important topic, with widely varying definitions of both disease and care seeking. There are also important geographical gaps in the published literature. Data on care seeking is available in the DHS (Demographic and Health Survey) [82] and MICS (Multiple Indicator Cluster Survey) [83] datasets and we would recommend that they were published in journal summary analyses. These would make the data more widely available, foster peer review, and encourage wider discussion of the findings and issues. Stepping up research efforts into interventions that aim to increase the ability of caregivers to recognise and seek appropriate care for childhood illness will be instrumental for designing evidence-based programmes and thus, further reducing childhood mortality in LMICs.

### Limitations of the Review

It is unlikely that this review has identified all relevant studies because we did not search the grey literature, the search strategy was carried out using English search terms only, and studies without an English abstract were not reviewed for inclusion in the review. The variations in study designs (e.g. prospective and retrospective designs), illness definitions and healthcare provider categories between studies affected the comparability of the studies’ results and also made a meta-analysis unfeasible. In particular, we decided to include studies reporting data on both hypothetical and actual care seeking behaviour because of the relatively low number of identified publications. There may, however, be considerable differences in care seeking rates reported in a hypothetical versus an actual illness scenario. Similarly, while education about illness and access to care is likely to have changed in many settings over the years, we did not exclude studies based on the year of data collection as this would have further reduced the already small number of publications identified for this review. A further weakness of the included studies arises from the fact that there is considerable overlap in the clinical presentation between the three diseases included in this review as well as other childhood illnesses. It is, therefore, likely that a proportion of participants in the included studies were misdiagnosed. Finally, given the wide variation in care seeking rates between studies and the significant geographical gaps in the literature, care should be taken in generalising this review’s findings to particular settings.

## Supporting Information

Table S1 **Details of search terms** for Medline and Embase (accessed via Ovid).
(DOCX)

Table S2 **PRISMA checklist.**
(DOCX)
