# Supplementary material for: The Recognition of and Care Seeking Behaviour for Childhood Illness in Developing Countries: A Systematic Review
Source: PLoS One. 2014 Apr 9;9(4):e93427. doi: 10.1371/journal.pone.0093427 (PMC3981715; doi:10.1371/journal.pone.0093427)
Supplement: Table S1 — Details of search terms for Medline and Embase (accessed via Ovid). (DOCX) [file pone.0093427.s001.docx]

## **S1: Details of search terms** for Medline and Embase (accessed via Ovid)^1^

*Diarrhoea:*

| Recognition | Care seeking |
| --- | --- |
| 1. exp Diarrhea/  2. diarrh?ea$.tw.  3. exp Gastroenteritis/  4. gastroenteritis.tw  5. enteritis.tw  6. gastritis.tw  7. dysentery.tw  8. or/1-7  9. recogni$.tw  10. perception.tw  11. validation.tw  12. or/9-11  13. 8 and 12 | 1. exp Diarrhea/  2. diarrh?ea$.tw  3. exp Gastroenteritis/  4. gastroenteritis.tw  5. enteritis.tw  6. gastritis.tw  7. dysentery.tw  8. or/1-7  9. exp Attitude to Health/  10. exp Health Behavior/  11. Illness Behavior/  12. Caregivers/  13. exp Parents/  14. treatment seek$.tw  15. care seek$.tw  16. health care seek$.tw  17. health behavi?r$.tw  18. health care behavi?r$.tw  19. care seeking pattern$.tw  20. health service utilisation.tw  21. or/9-20  22. 8 and 21 |

*Malaria:*

| Recognition and Care seeking |
| --- |
| 1. exp Caregivers 2. exp Mothers 3. exp Patient Acceptance of Health Care 4. exp Health Knowledge, Attitudes, Practice 5. exp Health Behavior 6. (care$ adj3 seek$).tw 7. (treat$ adj3 seek$).tw 8. recogni$.tw 9. exp Malaria 10. exp Fever 11. or/1-8 12. 9 or 10 13. 11 and 12 |

*Pneumonia:*

| Recognition | Care seeking |
| --- | --- |
| 1. exp Pneumonia/ 2. exp Respiratory Tract Infections/ 3. pneumonia$.tw 4. respiratory tract infection$.tw 5. acute respiratory infection$.tw 6. ARTI.tw 7. acute respiratory tract infection$.tw 8. or/1-7 9. recogni$.tw. 10. perception.tw. 11. 9 or 10 12. 8 and 11 | 1. exp Pneumonia/ 2. exp Respiratory Tract Infections/ 3. pneumonia$.tw 4. respiratory tract infection$.tw 5. acute respiratory infection$.tw 6. ARTI.tw 7. acute respiratory tract infection$.tw 8. or/1-7 9. exp "Patient Acceptance of Health Care"/ 10. Health Knowledge, Attitudes, Practice/ 11. exp Health Behavior/ 12. Caregivers/ 13. treatment seek$.tw 14. care seek$.tw 15. health care seek$.tw 16. health behaviour.tw 17. health behavior.tw 18. care seeking pattern$.tw 19. or/11-18 20. 8 and 19 |

^1^ Limits for all searches were 1) children aged less than 5 years, and 2) abstract available in English.
